# Diagnosis and management of fecal incontinence in children and adolescents

**DOI:** 10.3389/fped.2022.1034240

**Published:** 2022-10-18

**Authors:** Zhe-Ying Shen, Jin Zhang, Yu-Zuo Bai, Shu-Cheng Zhang

**Affiliations:** ^1^ Department of Pediatric Surgery, Shengjing Hospital of China Medical University, Shenyang, China; ^2^ Department of Pediatric Orthopedics, Dalian Women and Children’s Medical Center, Dalian, China

**Keywords:** fecal incontinence, anorectal malformation, anorectal manometry, biofeedback, children, colonic transit time, radiofrequency energy delivery, sphincteroplasty

## Abstract

Fecal incontinence (FI) is a commonly occurring disease of high concern. It is characterized by voluntary and involuntary defecation in children and adolescents. It is not only a physical disease but also a psychological and behavioral disorder. FI poses a serious burden on individuals and their families and therefore has become a social problem. Unfortunately, the management of FI among children is still a challenge because the etiology varies widely. Constipation has been found to be the most common cause, while sphincter dysfunction and neurogenic abnormalities may also play a role. Currently, no consensus guidelines exist, and the criteria for selecting optional methods remain unclear. It is therefore necessary to improve the efficacy of diagnosis and management strategies of FI in children. This review focused on the classification and etiology, discussed the diagnosis and management methods of FI in children and adolescents, and aimed to guide future studies.

## Introduction

Fecal incontinence (FI) is defined as the voluntary or involuntary defecation in an inappropriate place during children's developmental age of 4 years or above. In children the rates of fecal incontinence vary from 1.6% to 4.4% (1). Boys are more likely to suffer FI (2). Only 37.7% of children aged 5–6 years and 27.4% of children sought medical care for incontinence (3). A cluster of physical and psychological problems occur in FI patients, including repeated infections, skin ulcers and scars, social anxiety disorder, behavioral problems, self-abasement or isolation, and other problems, which lead to guilt and embarrassment (4–6). Currently, no consensus guidelines for diagnosis and management strategies of FI in children and adolescents exist, and the criteria for selecting optional methods had to learn from the practice guidelines in adults which could not cover all aspects of FI in children. Therefore, it is imperative to improve the efficacy of diagnosis and management strategies of FI and to pay attention to the physical and mental development and behavioral status of such children. In this review, the etiology, diagnosis, and treatment of FI were summarized to guide future studies.

## Classification and etiology

FI in children is classified into two major groups: functional and organic. The largest group consists of functional FI, which is further classified into functional retentive FI (FRFI) and functional non-retentive FI (FNRFI) according to Rome II criteria (7). FRFI is the most common type of FI with a proportion of at least 80%. Most FRFIs are caused due to functional constipation, while FNRFI is a clinical diagnosis based on medical history and physical examination (1). The pathophysiological grounds of FNRFI remain unknown. To date, it is considered to be the result of several factors, including young age, a positive family history, male sex, and important life events such as the birth of a younger sibling, parental discord, a change in living conditions, and others (8). The causes of FI in children are different from those in adults (9). Congenital malformations are common in children and adolescents, while tumor, trauma, and inflammation are common in adults. Behavioral disorders such as autism, attention-deficit hyperactivity disorder, and affective development disorders are also common causes of FI in children. According to the etiological classification, FI in children and adolescents may be associated with constipation, sphincter defects, and neurogenic abnormalities. Such different pathogeneses render the diagnosis and treatment for children and adults different (Table 1).

**Table 1:** The potential diagnosis and management process of FI in children and adolescents.

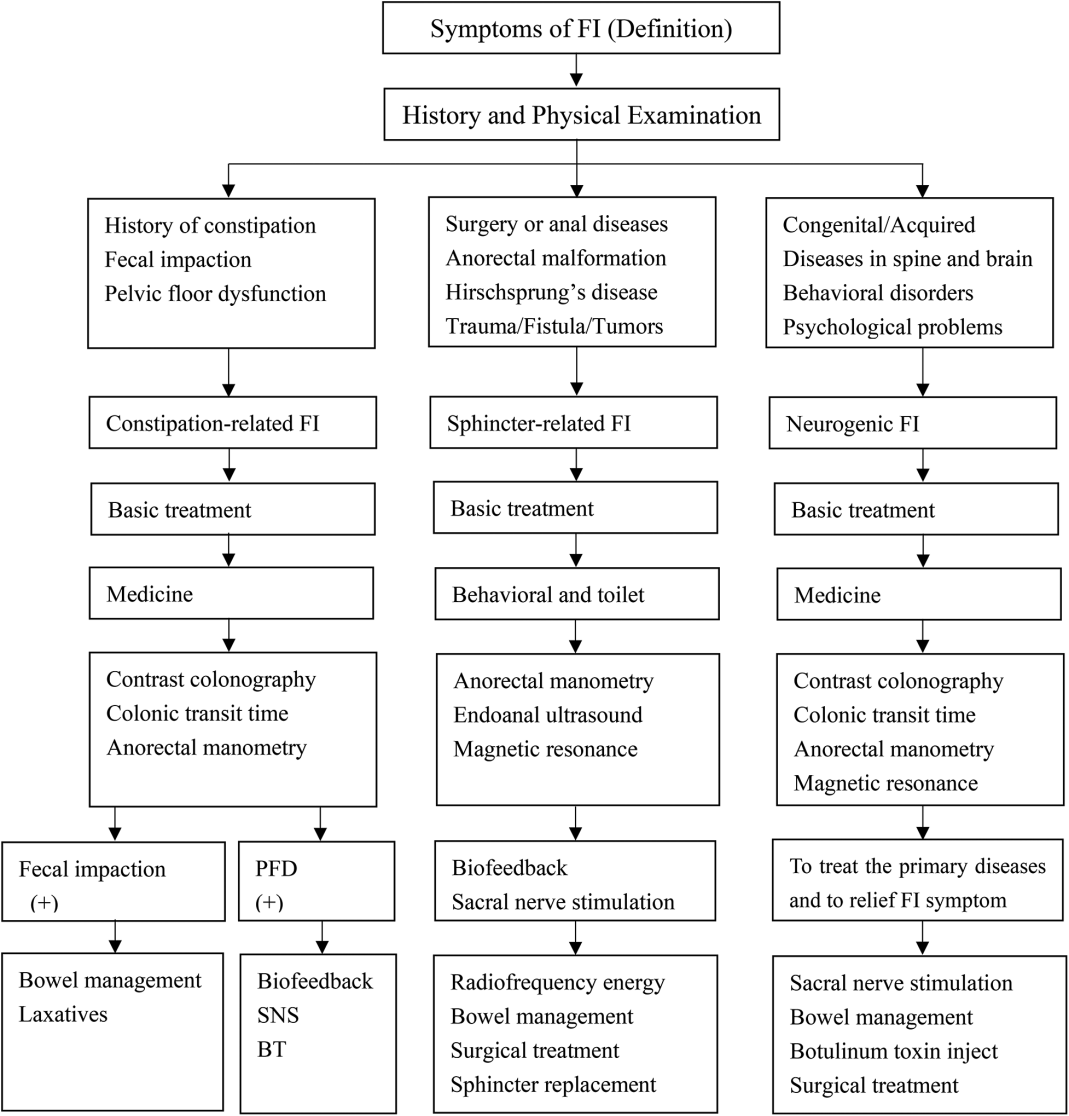

### Constipation-related FI

The most frequent causes of childhood constipation are improper toilet training and withholding behavior (5). Young toddlers experiencing painful defecation, such as from hard stools, have a natural instinct to retain their feces. These kids constrict their anal sphincters and gluteal muscles to withhold feces, which triggers a series of physiological changes that increase stool retention and cause both rectum and sigmoid colon dilation as well as the production of a big fecal mass in the rectum. Retained fecal matter finally becomes excessively stiff and difficult to be pushed by the high amplitude propagatory contractions as the water is absorbed *via* the intestinal mucosa. Due to bacterial activity, the stools in the upper portion of the fecal mass become liquid, seep over the distal hard fecal mass, and exit the anal sphincter, which results in FI. Causes such as fiber intake, food intolerance, and fecal impaction can cause constipation and eventually lead to FI in children.

### Sphincter-related FI

The internal anal sphincter, external anal sphincter, and puborectalis muscle are all involved in the anal canal closure function (10). The external anal sphincter is the most powerful part of the mechanism that closes the anal canal when it contracts. Since it produces tone and is in charge of more than 70% of resting anal pressure, the internal anal sphincter is crucial in the preservation of fecal continence. Along with the anal sphincters, the puborectalis muscle also contributes to the pelvic barrier, maintaining the apposition of the anal canal's walls, which, in normal conditions, inhibits fecal leakage with accurate sensory discrimination. Weakness or absence of any of these muscles will affect defecation continence. The sphincter-related FI or anal FI, often involves anal sphincter dysfunction resulting from anorectal malformation, anal sphincter trauma, Hirschsprung's disease (HD), anal abscesses/fistula, inflammation, and tumors (11).

### Neurogenic FI

The neurogenic FI is a specific type of FI reported in both children and adolescents, which can be either FRFI or FNRFI. The neurogenic abnormalities that account for FI can be congenital or acquired diseases of the spinal cord and brain. The diseases of the spinal cord include myelomeningocele, myelodysplasia, spina bifida, sacral agenesis, tethered spinal cord, and spinal cord malformations, which are all congenital. The acquired factors for neurogenic FI include acquired spinal cord injury, myelitis, and tumor(s) in the spinal cord (9, 12). The diseases of the central nervous system are also the main causes of FI in children, and almost all the diseases of the brain can partly trigger the symptoms of FI (13–16) (Table 1).

## Diagnosis of FI in children and adolescents

### Medical history and physical examination

The key information on the diagnosis and management of FI in children and adolescents are listed in Table 1. The medical history and outcomes of physical examination are mainly considered to determine the causes and risk factors (17). A thorough medical history includes gathering information on the duration and severity of symptoms and complications (18). FI can be caused by one or more factors such as the anorectal malformation, perineal trauma, obesity, low activity, chronic diarrhea, diabetes, neurological diseases, abscess, inflammation, anal fistula, sexual abuse, anal surgery, urinary incontinence, and drug use. A detailed physical examination is imperative in the assessment of FI. A systematic physical examination involves abdominal, anorectal, and neurological assessments. During a perianal inspection, doctors should examine the exits for any anatomic abnormalities, perianal feces, fissures, hemorrhoids, scars, and erythema. Digital rectal examination can provide important information about rectal stool masses, anorectal sensation, and sphincter tone (18). Examination of the lower spine can provide information about the height and deviation, if any, from the midline of the gluteal sulcus; it can also reveal abnormalities of the spine such as occult spinal dysraphism.

### FI score/scale

An appropriate scoring system is essential to both predict the severity of the disease and quantify the effect of treatment. Currently, many FI scales have been developed for adults (19, 20), which are not suitable for children because of the differences in the etiology. Therefore, a number of pediatric FI scoring systems were also developed, including Holschneider, Kricknebeck (21), Pena (22), and Rintala scores (23) (Table 2). For comparison, soiling and fecal frequencies and consistency were used in all these scores. The Holschneider score did not include the item “constipation,” but emphasized the need for its treatment. Kricknebeck and Pena's entries were simple and easy to use. The Rintala scoring system was the most comprehensive one, covering the contents of Kricknebeck and Pena, while also including the items related to “social problems.” It is currently the most widely used because it reflects the psychosocial problems to a certain extent. Researchers should choose appropriate scoring systems according to their own purpose and relevance. If a study emphasizes the importance of treatment, the Holschneider score is recommended, whereas the Rintala score is used when a design emphasizes quality of life(QoL) or psychosocial aspects (4). All the aforementioned scoring systems are meant for assessing sphincter dysfunction, but FI in children is mostly constipation related. Therefore, the scoring systems must be used to evaluate the constipation status in children, of which the Rome IV criterion is the most commonly used (24, 25).

**Table 2:** The common FI scores in children and adolescents.

Symptoms	Grade	Scores
**A. Holschneider questionnaires**		
Frequency of defecation	Normal (1–2/day)	2
	Often (3–5/day)	1
	Very often	0
Fecal consistency	Normal	2
	Soft	1
	Liquid	0
Soiling	No	2
	Stress	1
	Constant	0
Rectal sensation	Normal	2
	Reduced	1
	No discrimination	0
Ability to hold back	Yes, for minutes	2
	Yes, for seconds	1
	No, not capable	0
Discrimination of stool consistency	Normal	2
	Reduced	1
	No discrimination	0
Need of therapy	Never	2
	Sometimes	1
	Always	0
**B. Pena's questionnaires**		
Voluntary bowel movements: feeling the urge, capacity to verbalize, and holding the bowel movement	Yes	1
	No	0
Soiling	Occasionally (once or twice per week)	2
	Every day, no social problems	1
	Constant, social problem	0
Constipation	Manageable by changes in diet	2
	Requires laxatives	1
	Requires enemas	0
Urinary incontinence	Mild dribbling or wetness day and night	1
	Complete incontinence	0
**C. Rintala's questionnaire**		
Ability to hold back	Always	3
	Problems less than 1/week	2
	Weekly problems	1
	No voluntary control	0
Feels/reports urge to defecate	Always	3
	Most of the time	2
	Uncertain	1
	Absent	0
Frequency of defecation	Every other day to twice a day	2
	More often	1
	Less often	1
Soiling	Never	3
	Staining less than once a week, no change of underwear required	2
	Frequent staining, change of underwear often required	1
	Daily, requires protective aids during day and night	0
Accidents	Never	3
	Fewer than once a week	2
	Weekly accidents, often requiring protective aids	1
	Daily, requires protective aids during day and night	0
Constipation	No constipation	3
	Manageable with diet	2
	Manageable with laxatives	1
	Manageable with enemas	0
Social problems	No social problems	3
	Sometimes (foul odor)	2
	Problems causing restrictions to social life	1
	Severe social and/or psychic problems	0
**D. Krickenbeck questionnaire**		
Voluntary bowel movements: feeling the urge, capacity to verbalize, and holding the bowel movement	Yes	1
	No	0
Soiling	No	3
	Occasionally (once or twice per week)	2
	Every day, no social problems	1
	Constant, social problem	0
Constipation	No	3
	Manageable by changes in diet	2
	Requires laxatives	1
	Requires enemas	0
**Must have ≥2 the following criteria for ≥ 1month:**	**Grade**	**Scores**
**E. Rome IV criteria for functional constipation in Children and adolescents**		
≤2 defaecations in the toilet per week	Yes	1
	No	0
History of painful or hard bowel movements	Yes	1
	No	0
History of retentive posturing or excessive volitional stool retention	Yes	1
	No	0
History of large diameter stools that can obstruct the toilet	Yes	1
	No	0
Presence of a large fecal mass in the rectum	Yes	1
	No	0
≥1 episode of fecal incontinence per week	Yes	1
	No	0

### Air-barium double-contrast colonography

Air-barium double-contrast colonography is mainly used to observe the morphology of the entire colon and identify the presence of congenital colon malformations, such as HD, in infants and young children. However, in older children, it is used for mainly observing colon dilation and fecal impaction. These indicators are helpful in determining the type of FI, whether it is overflow incontinence caused by constipation or sphincter-related FI. Constipation and the severity of constipation-related FI can also be determined (26). It has advantages such as ease of performance, low cost, and ready availability (27, 28). Air-barium double-contrast colonography has to be preferred to CT because of its lower radiation dose and over MRI because of its lower cost. Therefore, air-barium double-contrast colonography can be performed in all the patients with constipation and FI.

### Colonic transit time

Colonic transit time (CTT) is routinely recommended to diagnose FI in children with constipation and neurogenic abnormalities. CTT is the most important method used for distinguishing between FNRFI and FRFI; relevant studies show that 90% of children with FNRFI have normal CTT results (8). Moreover, the results of CTT tests are critical for identifying the slow colonic transit, outlet obstruction, and various other reasons. Gyung Lee et al. demonstrated that a prolonged CTT value indicated severe constipation. Children with abnormal CTT results respond poorly to medication as compared with those with normal CTT results (29). CTT is also widely used in neurological FI. Studies demonstrated that normal transit was the most frequent subtype in the non-soiling group, as against slow transit in the soiling group (30). Vande Velde et al. showed that constipation was observed in 10 of the 38 children with spastic CP, and difficult defecation was found in other 19 children. All the children with FI displayed an abnormal segmental CTT in at least one segment of the colon. These results suggested that CTT could be used as a quantitative measure for constipation, which could distinguish between slow and fast transit encopresis (14).

### High-resolution anorectal manometry

Anorectal manometry is a test that measures the neuromuscular function of the rectum and anal sphincter complex (31–33). The latest internationally recognized examination procedure is high-resolution anorectal manometry (HR-ARM). Lusine Ambartsumyan et al. compared intra-anal pressure profiles between children with anorectal malformation and controls using HR-ARM and determined the association between manometric properties. They found that children with anorectal malformation had abnormal sensation and significantly lower pressures longitudinally across the anal canal, and believed that HR-ARM was a key component in the evaluation of FI in children with anorectal malformation (34). Annalisa Alessandrella et al. demonstrated that HR-ARM pressures under resting and squeezing conditions in children with constipation and FI were lower than in children with constipation without FI, particularly in anteroposterior quadrants. Compared with children without lower gastrointestinal symptoms, children with or without FI exhibited lower pressures and higher values of rectoanal inhibitory reflex (35). Tran reported that the prevalence of FI and constipation was 22.6% and 13.2%, respectively, in the enrolled 53 children following surgery for HD. The values of resting anal pressure and the maximum tolerated volume in incontinent patients were significantly lower than those in continent patients (36). Similar results were also reported by other researchers (37). A standardized protocol of HR-ARM can also characterize FI from dyssynergic or disordered defecation and other neuromuscular and sensory problems (9, 38, 39). Therefore, HR-ARM provides useful information that can guide better management in patients with defecation disorders, and it can be used in all kinds of FI in children and adolescents.

### Endoanal ultrasound examination

Endoanal ultrasound (EUS) is used to assess the condition of anal sphincter due to alloplasia, injury, surgery or perianal abscess. The correlation between sphincter morphology and FI symptoms can be determined by quantitative analysis of the sphincter size. EUS is considered a diagnostic tool for anal incontinence. Wang et al. assessed the postoperative anorectal anatomy and function using EUS in 47 children who underwent posterior sagittal anorectoplasty or transperianal anorectoplasty for anorectal malformations. They found significant differences in the thicknesses of the internal and external sphincters between the patients and healthy controls, and evidenced that the posterior sagittal anorectoplasty procedure could preserve the internal sphincter and anal functions in children with intermediate and significant defects (40). In another study, Parente et al. performed an EUS-assisted autologous intersphincteric injection of autologous microfragmented adipose tissue in four patients with FI following surgery for anorectal malformation. A significant improvement in bowel function FI scores were found (41). Meanwhile, the EUS performance needs a specific rectal 360° probe and cooperation between doctors and patients. These technical limitations in some hospitals and poor compliance in some children restrict the use of EUS in children.

### Magnetic resonance examination

The diagnostic purpose of MR varies based on the target organs. Head MR is performed mainly to rule-out the diseases of the central nervous system, including hypoxic-ischemic encephalopathy, developmental encephalopathy, white matter lesions, brain parenchymal lesions, and myelin lesions, cerebellar midbrain, and others. The lumbosacral MR is used to identify myelopathy, including tethered cord, myelomeningocele, spinal cord tumors, and so on. Sphincter MR is more suitable for observing the nature of the anal sphincter such as the shape, thickness, directions, and position of the anal sphincter complex and its location on the pelvic floor. MR examination has a high clinical value in the diagnosis of anorectal malformation. It can help determine the anal atresia type, display the presence and running of the fistula, evaluate the perianal muscle development and other systems' malformations, and finally provide a reliable diagnostic basis for surgical program and prognostic assessment (42). The role of MR is similar to that of EUS in some aspects. However, the sphincter MRI can clearly demonstrate the sphincter pattern, the position of the sphincter on the pelvic floor, and several indicators that cannot be detected by EUS. Also, it has low technical requirements and can be performed in all hospitals.

### Pudendal nerve terminal motor latency

Pudendal nerve terminal motor latency (PNTML) measures the neuromuscular integrity between the terminal portion of the pudendal nerve and the external anal sphincter. Delayed response is associated with pudendal neuropathy, which can contribute to FI. Some disagreement exists as to whether this diagnostic maneuver has any benefits (43). Cheong et al. demonstrated that prolonged PNTML test was not significantly associated with most anorectal manometry findings or with subjective measurements of the severity of FI (44), and only 34% of patients with idiopathic FI displayed pudendal neuropathy (44). In addition, some studies evidenced a lack of correlation between prolonged PNTML and anal manometric measurements. PNTML can only be used to help identify the etiology of FI in patients with intact sphincters and normal manometry pressures (45). Although pudendal neuropathy is a potential cause of FI, the measurements of PNTML have not been found to be helpful in FI detection.

## Management of FI in children and adolescents

### Basic treatment

The basic treatment is the first-line treatment option of FI in children and adolescents. It has been reported that 22%–54% of patients with FI achieve a high QoL through diet, toilet training, and cognitive behavioral therapy (5).

A well-balanced diet should be encouraged, which includes fruits, vegetables, and plenty of water, and constipating foods such as cheese and white rice should be limited. Food components that lead to fecal urgency or diarrhea should be paid attention to. In children and adolescents, lactulose and oligosaccharides are used for treating constipation, but they are known to have bad effects on FI. Dietary fiber can promote intestinal peristalsis, soften stools, and reduce the occurrence of constipation; they should be administered individually according to the type of FI. Age plus 5 g (e.g., 8 g/d at age 3, 15 g/d at age 10, and 25 g/d at age 20) is the formula used to determine the minimum daily fiber intake (g/day) for children and adolescents from 3 to 20 years of age. Thereafter, adult standards of 25 to 35 g/d should be followed (46). Patients with constipation-related FI are suggested to take more dietary fiber (9, 47), whereas the fiber intake should be decreased in patients who have impaired sphincter function. Adequate fluid intake optimizes the effect of osmotic laxatives and fiber; it is also necessary for overall bowel health. The recommended daily fluid consumption for a typical kid is as follows: 5–10 kg: 500–1,000 ml; 10–20 kg: 1,000–1,500 ml; 20–30 kg: 1,500–1,750ml; 30–40 kg: 1,750–2,000 ml; 40–50 kg: 2,000–2,250 ml; >50 kg: 2,250–2,500 ml (9).

Cognitive behavioral therapy is psychotherapy proved to be effective for FI. It involves cognitive and behavioral therapies. The toilet training recommended for FNRFI is also a type of cognitive behavioral therapy, which can be enhanced with simple techniques such as praise, rewards, and token systems (8). An appropriate toilet program is considered the first important element. It helps children take advantage of the gastrocolic reflex and facilitates defecation (9). It is advisable for a child with functional FI to sit on the toilet three times per day, ideally after meals, for 5–10 min. To get the most out of the program, proper sitting, relaxed posture, and foot support when necessarily must be taught. Children with FNRFI should not only use the restroom after meals but also train right away in the afternoon after returning from school. This is due to the fact that most of these kids experience fecal incontinence between 3 and 6 pm. For many noncompliant patients, administering this toileting program may be a serious problem, especially for children with externalizing behavioral disorders such as oppositional defiant disorder and attention-deficit hyperactivity disorder. Approximately 30% of the children with functional FI have significant emotional and behavioral problems (48). In these children referral to a clinical psychologist is helpful and increases treatment success. Evidence shows that behavioral interventions improve continence symptoms in children with FI associated with constipation (49). In the case of behavioral problems, behavioral therapy should be considered. Besides, abdominal massage has been used beneficially by 22%–30% of patients with neurogenic bowel dysfunction (NBD). In a study of 24 patients with spinal cord injuries, abdominal massage in addition to the standard bowel program led to a significant reduction in CTT, abdominal distension, and FI, while increasing the frequency of defecation (50).

### Medicine

Medicine is also a commonly recommended treatment for FI. In children, constipation-related FRFI is the most common type of FI. Laxatives have been considered appropriate because they remove fecal impaction in the colon and rectum. A systematic review of laxatives has shown that cellulose increases the frequency of defecation (51). The main aim of pharmacotherapy for constipation-associated FI is to remove fecal impaction and maintain soft stools. It can be augmented by the use of laxative suppositories. Glycerin(e)/glycerol and bisacodyl are the commonly used suppositories, with the former mild enough to be used in infants, but often ineffective in older children with NBD. The latter is a stimulant laxative that has either hydrogenated vegetable oil or polyethylene glycol (PEG) as a base. Polyethylene glycol is shown to be effective in both disimpaction and preventing the reaccumulation of stools. Children treated with polyethylene glycol had fewer FI episodes, less frequent re-impaction, and significantly lower medical costs. A systematic review proved that polyethylene glycol was more effective in treating constipation than other osmotic laxatives. Currently, PEG is the first choice for treating constipation-associated FI in children (52). Dheivamani proved that PEG 3,350 was superior to lactulose in the maintenance therapy of young children with functional constipation (53). Polyethylene glycol has been routinely recommended in the treatment of constipation-related FI in NASPGHAN/ESPGHAN guidelines.

Medical management with laxatives has been successful in patients with sphincter-related FI. Wood conducted a retrospective study in 222 children with anorectal malformation. The use of laxatives in these patients led to a good prognosis and helped them empty their rectum and colon effectively. They demonstrated that the patients could be treated with either laxatives or enemas depending on whether they had their own bowel control (54).

There is no specific evidence for the use of probiotics in children with Neurogenic FI. However, the use of a probiotic may be taken into account for a general improvement in gut health and microbial variety. High-quality data exist in the form of several RCTs confirming the beneficial effect of laxatives in individuals with NBD. In one RCT including pediatric NBD, polyethylene glycol (PEG)/macrogol was shown to be superior than lactulose, leading to higher bowel frequency (*p* < 0.01) (55).

### Biofeedback

In recent years, studies have increasingly confirmed that pelvic floor dysfunction is responsible for childhood FI. The pathophysiological basis suggests that biofeedback can be used as a potential treatment for pediatric FI. Random and nonrandom trials showed that 64%–89% of cases were relieved of the FI symptoms through biofeedback training. Nader et al. reviewed the medical records of 23 children with FI treated with biofeedback. About 83% of the children were doing well after biofeedback without relapse (56). A retrospective study with 46 children with FI following surgery for HD revealed that biofeedback training provided satisfactory results in that the FI symptoms disappeared in 84.78% of patients (31). Biofeedback can be used not only for FI caused by constipation, but also for other types of FI. Amy Tremback-Ball et al. found biofeedback to be a beneficial treatment for children with dysfunctional voiding and functional FI (57). Caruso et al. evaluated the biofeedback response in 25 children with FI treated for anorectal malformation using a clinical score, anorectal manometry, and pelvic MR. They found the overall response to biofeedback to be excellent in 44%, discrete in 40%, and poor in 16%. The manometry can evaluate the potential sphincter recovery after biofeedback (33). FI in children can also result from pelvic floor dysfunction or dyssynergic defecation, which can be both myogenic and neurogenic. Several previous studies suggested a long-lasting beneficial effect of biofeedback in children with FI secondary to myelomeningocele. In more than 50% of these children, FI symptom disappeared without the need for enemas or suppositories (9). Hence, biofeedback is a beneficial treatment for children with FI.

### Sacral nerve stimulation

Sacral nerve stimulation (SNS) has gained wide acceptance among doctors for its ability in urinary and FI treatment because it is thought to modulate the function of the bowel, bladder, and/or pelvic floor (58). Many studies confirmed that SNS was effective in treating FI that did not respond well to conventional treatment, and for constipation, with high patient satisfaction (59). Lu et al. evaluated the long-term efficacy of SNS in 25 children with constipation-associated FI. The symptoms of FI decreased from 72% to 20% and proved SNS to be a promising and durable treatment for children with FI (60). Similar trials for SNS in treating refractory constipation and FI were also reported by other authors (61). Lecompte examined transcutaneous SNS in four patients with anorectal malformation and found that two of them were cured. Their Wexner scores decreased from 13 to 5% and 75% of them stopped using antegrade enemas, while 50% reported spontaneous defecation (62). Therefore, the efficacy and safety of SNS in childhood FI have been deeply studied.

### Radiofrequency energy delivery

Radiofrequency energy delivery controls the application of radiofrequency energy to the anal sphincter complex and results in collagen deposition and tissue remodeling. This intervention was approved by the Food and Drug Administration in 2002 for treating FI that failed conservative treatments. Current practice guidelines state that the application of temperature-controlled radiofrequency energy to the sphincter complex may be used to treat FI (17). Frascio et al. assessed the results of the radiofrequency procedure in FI and found that both FI and QoL scores improved significantly following the treatment (63). However, Omar Vergara-Fernandez reported the opposite result. According to his findings, radiofrequency therapy did not display effective long-term results (64). All the current studies were conducted with a small number of patients and a short follow-up time. Hence, a large-sample randomized controlled trial is needed.

### Bowel management

Bowel management can offer a significant improvement in patients with NBD, constipation, and anorectal malformation (65). In general, two ways of bowel management exist: antegrade continence enema (ACE) and retrograde colonic enema (RCE). ACE through appendicostomy is also called malone antegrade continence enema (MACE), which has been reported as a very useful technique to resolve FI or constipation in children. ACE has been evidenced to be useful in the management of NBD, which is characterized by chronic constipation and/or FI; ACE *via* cecostomy or MACE had similar continence outcomes (66). A retrospective review of 6–18-year-old children with FC and FI treated with either ACE or SNS showed that SNS was more effective against FI and ACE in improving the stool frequency and soothing abdominal pain (67). Another comparative study by Born et al. reported that MACE might be more attractive than cecostomy tube in avoiding repeated procedures and radiation exposure (68). Gomez-Suarez et al. also reported similar results, in that they compared cecostomy tubes with MACE in the treatment of refractory constipation and FI. The results demonstrated that both constipation and colonic motility improved (69–71). Nowadays, ACE has become the most accepted procedure in treating children with constipation and FI (72). However, the downside of ACE procedure is the requirement of surgery. Hence, RCE becomes the mainstay of conservative treatment. King et al. confirmed the effectiveness of RCE in the management of FI in children (73). However, RCE does not conform to physiology because of the opposite transmission direction. Thus, the effects may be not as effective as those of ACE with prolonged treatment course.

### Botulinum toxin injection

Botulinum toxin(BT) injection into the anal sphincter appear to be an effective treatment for pediatric patients with constipation-associated FI and neurogenic FI (74, 75), which is believed to temporarily chemically paralyze the anal sphincter, changing the patient's bowel habits and alleviating symptoms (76). Kajbafzadeh et al. conducted a prospective study to evaluate the efficacy of botulinum toxin A in treating children with myelomeningocele and declared it to be completely successful in eight patients (53%) and moderately successful in two (13%) for the alleviation of symptoms. They concluded that botulinum toxin A appeared to be a safe, minimally invasive procedure for the management of NBD in children with myelomeningocele (75). Similar results were also reported by other researchers in studies on constipation-associated FI (74). However, evidence regarding the efficacy and safety is still lacking for this procedure. A few complications were reported, including urinary incontinence, pelvic muscle paresis, perianal abscess, pruritus ani, and rectal prolapse (74). Adverse effects of BT injections range from transient incontinence to anal pain and muscle fatigue. The drawbacks of botulinum toxin injection are the necessity of general anesthesia and the high costs. Botulinum toxin injections may be an alternative approach for managing patients with FI. The efficacy, safety and appropriate patient population for botulinum toxin injection in FI remains unknown. More data are needed to better determine the role of botulinum toxin in the management of FI.

### Surgical treatment

Bowel resection has been proposed for selected cases of functional constipation and/or FI following failed conservative treatment. The outcomes in these children were reported to be favorable in most of the cases (77, 78). However, bowel resection does not play a role in NBD apart from occasional limited resections during colostomy. Colostomy is a bowel diversion that produces similar or even superior outcomes in selected patients in terms of QoL compared with conservative bowel management strategies in NBD. Early colostomy after spinal cord injury has also been shown to improve the independence and acceptability of intestinal management (79).

Anal sphincteroplasty is conducted when the integrity of the sphincter is broken. In children, the disruption of the circumferential anatomy of the anal sphincter is mainly caused by cloacal malformation and trauma. Anal sphincteroplasty is an effective way for sphincter-related FI. Studies have shown that the remission rate of FI after anal sphincteroplasty can be as high as 75%–86% (80). In a few serious cases, the sphincter is very weak and nearly missing, and *in situ* local sphincter repair is ineffective. Another operation is needed to transfer the gracilis or gluteus maximus around the anal canal and replace or strengthen the sphincter function. Shi et al. compared the clinical effect of graciloplasty using two different gracilis encircled loops for the treatment of FI following anoplasty for imperforate anus and found gracilis neosphincter to be an efficient method for patients with FI (81). Although anal sphincteroplasty is effective to a certain extent, secondary or multiple sphincter reconstruction and external sphincter folding are not recommended. Anal sphincteroplasty is conducted only in sphincter-related FI, but not in constipation-related FRFI and neurologic FI.

### Sphincter replacement strategies

Sphincter replacement strategies (SRS) serve as a kind of replacement treatment for severely defective natural sphincter. The existing studies are all retrospective, and most of them reported satisfactory efficacy. SRS is generally recommended for sphincter defects, but not for neurologic incontinence. The injection of bulking agents around the anal canal can effectively reduce the frequency of involuntary FI and relieve symptoms, and can therefore be used in the treatment of sphincter-associated FI (41, 81, 82). Although increasing evidence shows the potential of SRS in FI, a few studies were performed in children. Hence, a large-sample randomized controlled trial is needed.

## Summary in the diagnosis and management process of FI

Management of FI among children is still a challenge because the etiology varies widely. Constipation was the most common cause, while sphincter dysfunction and neurogenic abnormalities may also play a role. Currently, no consensus guidelines exist, and the criteria for selecting optional methods had to learn from the practice guidelines in adults (83). It is therefore necessary to improve the efficacy of diagnosis and management strategies of FI in children. Base on the documented literature, the diagnosis and management process of FI should be carried out in the following diagram (Table 1).

## Conclusions

FI in children and adolescents can be associated with constipation, sphincter defects, and neurological diseases. The treatment program should be individualized following a comprehensive evaluation of neurological, colonic, and sphincter function.
